# Tool for the assessment spiritual care after the COVID-19 pandemic: a sequential exploratory study

**DOI:** 10.4314/ahs.v22i3.58

**Published:** 2022-09

**Authors:** Zahra Bostani Khalesi, Mohsen Pourmohammad

**Affiliations:** 1 School of Nursing and Midwifery, Guilan University of Medical Sciences, Rasht, Iran; 2 Department of Nursing, Langroud School of Nursing and Midwifery, Guilan University of Medical Sciences, Rasht, Iran

**Keywords:** COVID-19, Spiritual care, Factor analysis

## Abstract

**Background:**

The present study aimed to develop a tool to assess spiritual care after the COVID-19 Pandemic.

**Materials and methods:**

This study is a mixed sequential (Qualitative-Quantitative) exploratory study. In the qualitative phase, through 14 in-depth semi-structured individual interviews with clerics, specialists in Islamic jurisprudence and principles, specialists in education and health promotion, and using the content analysis approach, tool items were designed. Purposeful sampling was performed with maximum diversity of experts and enthusiasts in the field of spiritual health.

**Results:**

Content analysis of the data obtained from interviews led to explaining the concept of spiritual care after the COVID-19 Pandemic in four main themes: spiritual care needs, spiritual care characteristics, outcomes of spiritual care, and the challenge of providing spiritual care. The average content validity index of the tool was 0.94. Exploratory factor analysis showed 4 factors that explained more than 62.83% of the variance. The correlation of spiritual cares scale score for COVID-19 Pandemic with spiritual care tool was (0.86, p <0.001).

**Conclusion:**

Spiritual care tool is a valid and reliable tool, with 38 items to assess the spiritual care after the COVID-19 Pandemic.

## Background

The novel corona virus has recently entered the pandemic phase as the cause of the Covid-19 disease and has had significant negative effects on the mental health and behavioral quality of individuals in the family and society^1^. This disease has been unique in recent years due to its extent and speed of transmission^2^. Corona virus infection is very different from other viral infections due to its different biological, social, and psychological dimensions and has affected the mental health of individuals^3^. Since one of the most important concerns of people in stressful situations due to coronary heart disease is mental health and achieving peace, hence, special attention should be pay to the spiritual care dimension^4^. According to research conducted by psychologists in various crises, it has been found that religion and spiritual care have a tremendous effect on mental health and the research of some world-famous psychologists emphasizes the positive effect of religious values, especially prayer on mental health^5^. They believe that spiritual health promotes positive emotions^6^.

Spiritual health is an aspect of health that brings mental relaxation and a sense of wholeness and well-being^7^. Of course, experts have expressed different definitions of spiritual health^8^. According to one definition, spiritual care can be consider as a balance between the physical, mental, social, and spiritual aspects of the individual, which is a process of progress towards a harmonious relationship that originates from the inner forces of the individual^9^. In another definition, spiritual care is having a sense of acceptance, positive emotions, morality, and a sense of positive interaction with a sovereign and superior divine power with others and oneself through a dynamic and harmonious process of cognition, emotion, and action^10^. In any case, spiritual care is at the top of Maslow's hierarchy of needs and reflects the quality of one's life in the spiritual dimension^11^.

Providing a single definition of spiritual care is difficult and challenging due to the interpretability of the two concepts on the one hand and the significant changes in their interpretations of “spirituality” and “health” on the other^12^. Therefore, despite the many studies that have been done in this field during the past decades, the lack of a comprehensive and acceptable definition of spiritual care is still observed^13^. Many studies do not distinguish between spirituality and religion, and others, although deeply inherent in the two concepts, do not equate them^14^.

In accordance with the existence of different perspectives on the concept of spirituality and spiritual health, various tools have been design and introduce to measure this dimension of health^15^. Some tools examine the mental health of individuals or a common theme between two areas rather than assessing their spiritual care^16^. It has also been effective in developing tools for measuring the spiritual care of various perspectives, including cultural contexts^17^. Therefore, in addition to the need to conceptualize spiritual care related to the cultural, economic, and social characteristics of society in the Corona period, the design of indigenous tools to measure spiritual care is one of the necessities of this field. Because it is inappropriate to pay attention to spiritual care without considering cultural contexts or the same spiritual culture as sowing seeds in the ground. Determining cultural compatibility is of particular importance in tool design^18^.

The corona crisis, due to its special nature, has caused people to become anxious, and a tendency towards spirituality seems to be essential for achieving peace14. However, because the care services provided during the epidemic period of the disease are more focus on the general dimensions of health, the spiritual care dimension of individuals may be neglect^19^. In order to benefit from the services of this dimension of health, it is necessary to provide a comprehensive and acceptable definition of it in this crisis^20^. However, despite much research on spiritual care over the past decades, searches of reputable databases have not found a comprehensive definition of spiritual care based on the characteristics of the Corona period^21^. There is no consensus on the structure of spirituality and consequently the concept of spiritual care8. On the other hand, assessing the spiritual care status of individuals after the manifestation of this virus in the first step requires providing a definition of spiritual care according to the characteristics of this course and then designing and testing tools that can assess the conceptual structures of the definition provided. Therefore, this study aims to develop and assess psychometric properties of a tool to assess spiritual care tool after the COVID-19 Pandemic.

## Method

The present study is a mixed sequential (Qualitative-Quantitative) exploratory study. In the qualitative part, the concept and dimensions of spiritual care after the corona pandemic were explained and in the quantitative part, the tool “spiritual care after the corona pandemic" was designed and psychometric. Qualitative data collection was performed using 14 in-depth semi-structured individual interviews with clerics, specialists in Islamic jurisprudence and principles, specialists in education and health promotion. Sampling was started purposive and continued until data saturation. Inclusion criteria were age over 18, mental ability to interview and answer questions, and ability to speak Persian. The interviews were typed face-to-face with permission to record (Sony VOR audio recording) and word-for-word. The guide to the interview questions was prepared by reviewing the texts and a preliminary study. In-depth semi-structured interviews began with a general question (What do you think is the meaning of spiritual care in Corona?). Each interview lasted between 60–45 minutes. Ethical obligations to protect the rights of participants were considered by the researcher, the purpose of the study was explained and the privacy of information, confidentiality, and the right to leave the study at any time was guaranteed. All participants participated in the study with written consent. Interviews were conducted and recorded at the specialist workplace. Software MAXQDA v.10 was used for data analysis. In order to examine the data of this study, the criteria of Cuba and Lincoln (1989) including validity, transferability 2, homogeneity, and validity were used^22^. The tool was scored based on a 6-point Likert tool, scoring 1 to 6 for “Agree Very Strongly”, “Agree Strongly”,” Agree”,” Disagree”,” Disagree Strongly” and, “Disagree Very Strongly” respectively. The total score of the tool is determined by calculating the average scores of the total items.

In the quantitative part of the study, the validity and reliability of the tool were assessed. Content validity, face validity, construct validity (exploratory factor analysis) and criterion validity (simultaneous) were used to evaluate the validity of the health needs tool. To determine the face validity of the tool, two methods of qualitative and quantitative face validity were used. The qualitative face validity of the tool was determined through face-to-face interviews with 20 men and women and a review in terms of difficulty, appropriateness, and ambiguity in the items. Only questions were acceptable in terms of face validity and were found suitable for further analysis if their impact score was higher than 1.5.

To evaluate the content validity of the Health Needs Questionnaire, the opinions of 20 faculty members of the seminary and the university (specialists in the field of spiritual health, spiritual, jurisprudence and Islamic principles, health education and promotion specialists) were used to determine the content validity index (Content Validity Index) Waltz and Basel determine the relevance, clarity, and simplicity of each item in the questionnaire in the 4-point Likert tool^23^. The content validity index score for each phrase is calculated by dividing the number of agreeing on experts for the Likert rank 3 and 4 by the total number of experts. Based on this index, all expressions are measured at the beginning. If the index obtained is 79% or higher, that phrase will be accepted^24^. Content validity was assessed qualitatively and quantitatively with the participation of 20 experts with knowledge and experience in tool design. In the qualitative method, 20 tool making experts were asked to comment on the tool items in terms of grammar, use of appropriate words, placement of phrases, and appropriate scoring. “Content validity ratio” and “Content validity index” were used to evaluate the validity of quantitative content. The reliability of the tool was confirmed by two methods of determining internal consistency and retest test. The tool used the Exploratory Factor Analysis method, which examines the internal relationship between variables, in order to discover the classes of variables that were most related to each other. The validity of the criterion (simultaneously) was assessed using the spiritual care tool (SWBS). Ellison developed the spiritual care tool in 1983 25. To determine the reliability of the Spiritual care tool after the Corona pandemic, two methods of internal consistency and reliability were tested. The internal consistency of this tool was assessed in two stages. First, before performing factor analysis and in a sample of 200 men and women, it was determined. In the second step, after performing factor analysis, Cronbach's alpha coefficient for each factor as well as the whole tool was calculated in a sample of 20 women and 20 men. Exploratory factor analysis, Cronbach's alpha calculation, intra-cluster correlation index, and Wilcoxon test were used for statistical analysis of the second part of the study. The University Ethics Committee with the number IR.GUMS.REC.1399.515 approved this study.

## Results

Content analysis of data from interviews with seminary and health professionals led to the explanation of the concept of spiritual care after the Corona pandemic in four main themes: spiritual care needs, spiritual care characteristics, outcomes of spiritual care, and the challenge of providing spiritual care. The total number of items in the tool in the first part of the study and after the end of the interviews and review was 147 items. The selected items were then examined in three sessions by the research team and the items that had merged concepts were merged and the initial tool consisted of 112 items were designed.

At the end of the content validity stage, the items of the tool were reduced from 112 to 97 items. The average tool content validity index (S-CVI) was 0.94. In evaluating the construct validity of the tool by exploratory factor analysis, the adequacy of sampling was assessed by Kaiser Meyer Olkin (KMO) test, which was 0.86. Then, in order to determine whether the obtained correlation matrix is significantly different from zero, and based on which factor analysis can be justified, Bartlett's sphericity test was used.

In the next step, after calculating the correlation matrix between the variables, the factors were extracted. Principal Component Analysis extracted factors embedded in the tool. In this study, Scree Plot and Eigen Value methods were used to determine the number of components of the Spiritual care tool after the corona pandemic. In addition, from the turning point of 0.40, it was considered, as the minimum factor load required maintaining the expression in the factors extracted from factor analysis. Initially, 21 factors with eigenvalues above 1 were identified, which together represented 82.14% of the variance. In order to simplify and interpret the factor structures, a tool was designed, and due to the low explanatory power of the end factors and considering the degree of agreement of the extracted factors with the concept and dimensions of spiritual care after the corona pandemic that was explained in this study. By limiting the extraction of agents to 4 factors and using the Varimax period, exploratory factor analysis was performed again. The Varimax period, which is orthogonal rotation (orthogonal rotation) is used to simplify and interpret the operating structures. Finally, 4 factors that represented a total of 48.22% of the variance were accepted, 42 phrases that could not reach the minimum factor load of 0.40 or had repetitive meanings were removed and the remaining 55 phrases were named in 45 constructs (sub tools). Spiritual care (10 items), Spiritual care provider characteristics (8 items), and spiritual care needs (19 items) were called 12.82, 12.47, 9.26, 8.27, and 5.24% of the variance, respectively (Table 1).

**Table 1:** Spiritual care tool after Corona Pandemic

	Statements	Agree Very Strongly	Agree Strongly	Agree	Disagree	Disagree Strongly	Disagree Very Strongly
**Outcomes of spiritual care**							
1	Patience						
2	Trusting God						
3	Hope						
4	Peace						
5	Reduce pain						
6	God-seeking						
7	Theology						
8	God-centered						
9	Knowing the purpose of creation and life						
10	Striving for spiritual excellence						
11	Improve the quality of life						
**The challenge of providing spiritual care**							
12	Weakness of religious beliefs						
13	Failure to provide spiritual care services						
14	Low knowledge of health care providers						
15	Resistance to religious issues						
16	Lack of specialized team						
17	Lack of access to quality services						
**Spiritual care provider characteristics**							
18	Positive attitude						
19	Respect for human dignity						
20	Understand and respect the religious values and beliefs of individuals						
21	Verbal skills						
22	scientific skills						
23	Communication skills						
**Spiritual care needs**							
24	Facilitate spiritual growth						
25	Spiritual support						
26	Creating a positive attitude						
27	Teaching spiritual self-care						
28	Empowering health care providers						
29	Provide spiritual care regardless of faith and religion						
30	Continue spiritual care after discharge						
31	Access to spiritual care services						
32	Attract the support of health policy makers						
33	Allocate resources						
34	Give proper priority to spiritual care						
35	Establish private centers providing spiritual care services						
36	Training of spiritual care specialists						
37	Integrating spiritual careinto care-treatment programs						
38	Designing a spiritual care system						

The results of the criterion validity of the designed tool showed a correlation between the scores of the Spiritual care tool after the corona epidemic and the Spiritual care tool (SWBS) (r = -1.74 and p <0.001). The reliability results of the tool showed an initial Cronbach's alpha coefficient of 0.90 (after examining the content and face validity index). In addition, Cronbach's alpha coefficient of the designed tool (after performing factor analysis) was 0.80–0.90 for the tool factors and 0.91 for the whole tool. The consistency of the tool was retested using the intracluster correlation index test (p <0.001, ICC = 0.972). It was used that the difference in scores in its two performances was not significant (p> 0.05).

## Discussion

The present study focused on the concept of spiritual care after the corona pandemic and based on group and individual interviews with key informants and by studying the available sources, the Spiritual care tool after the corona pandemic was designed and then its psychometric analysis was performed.

According to a review study conducted in 2011, among the 35 valid assessment tools of treatment designed to measure spiritual health, the tools of chronic diseases-spirituality and the spiritual care tool provided by Ellison have been the most used ones in spiritual care research^25^.

Assessing the construct validity of the spiritual care tool after the corona pandemic with factor analysis showed that the tool was a four-factor tool including spiritual care needs, spiritual caregiver characteristics, outcomes of spiritual care, and the challenge of providing spiritual care.

The results of this study showed that the spiritual care tool after the corona epidemic could be used as a valid and reliable tool for gather the necessary information about spiritual care to be used after the emergence of this pandemic. The average content validity index of the tool was 0.90. Exploratory factor analysis showed that the designed tool had four factors. Correlation of spiritual care tool score after Corona pandemic with spiritual care tool was (0.86, p <0.001). The content validity of the Spiritual care tool was assessed after the corona pandemic based on the opinions of experts. Examining the validity of the content of the tool by experts is one of the best ways to gather evidence in support of a tool^26^. In line with this study, there are similar studies that confirmed the validity of the content of their tool by using the opinions of a group of experts and specialists.

Measurement of internal consistency of the tool showed Cronbach's alpha coefficient of the tool 0.90 and measuring the stability of the tool showed the intra-cluster correlation index at two-week intervals (p <0.001, 0.972). The existence of reliability in the tool is one of the most important criteria that shows the quality of the tool. The Spiritual care tool had acceptable internal consistency and stability after the corona pandemic. Other studies in the field of tool design the field of spiritual care has been done, this method was used to confirm the reliability of the designed tool^27^.

One of the differences between this tool and some of the tools that have been introduced so far is its reliance on the concept of spiritual care after the corona pandemic in Iranian culture. One of the characteristic features of this difference may be the existence of “spiritual care needs” as one of the components. Health needs in this period. Items such as “Spiritual care provider characteristics”, “Outcomes of spiritual care”, and “The challenge of providing spiritual care” show that people after the emergence of this pandemic part of their health needs in connection with the eternal source. They seek revelation and in order to quench their spiritual thirst, they need religious truths to be explained to them in a strong and reasoned manner.

## Conclusion

Based on the results of this study, the Spiritual care tool after the corona pandemic was designed based on the concept of spiritual care after the corona pandemic in the Iranian culture. The tool is designed based on the understanding of the concept of spiritual care after the corona pandemic from the perspective of field experts and health professionals and after examining their experiences through in-depth and qualitative research techniques. Spiritual care is appropriate after the Corona pandemic in Iranian society. Therefore, considering the lack of valid and reliable tools and in accordance with the cultural conditions of Iran to assess spiritual care after the corona pandemic, the present study can be useful in achieving the above goals.

Testing a tool designed on Iranian society makes the generalizability of the results difficult for other countries. In addition, the sample size in this study did not allow a more accurate study of spiritual care status in different population groups. However, the results obtained from the initial study indicate the desired validity and reliability of the comprehensive tool for measuring spiritual care after the corona pandemic in Iranian society. Additional studies are recommended to assess spiritual care in different populations and regions of the country.
